# A clear urban–rural gradient of allergic rhinitis in a population-based study in Northern Europe

**DOI:** 10.3402/ecrj.v3.33463

**Published:** 2016-11-25

**Authors:** Stine Holmegaard Christensen, Signe Timm, Christer Janson, Bryndis Benediktsdóttir, Bertil Forsberg, Mathias Holm, Rain Jogi, Ane Johannessen, Ernst Omenaas, Torben Sigsgaard, Cecilie Svanes, Vivi Schlünssen

**Affiliations:** 1 Department of Public Health, Section for Environment, Occupation and Health, Danish Ramazzini Centre, Aarhus University, Aarhus, Denmark; 2 Department of Medical Sciences: Respiratory, Allergy and Sleep Research, Uppsala University, Uppsala, Sweden; 3 Faculty of Medicine, University of Iceland, Reykjavik, Iceland; 4 Division of Occupational and Environmental Medicine, Department of Public Health and Clinical Medicine, Umeå University, Umeå, Sweden; 5 Department of Occupational and Environmental Medicine, Sahlgrenska University Hospital, Gothenburg, Sweden; 6 Lung Clinic, Tartu University Hospital, Tartu, Estonia; 7 Centre for Clinical Research, Haukeland University Hospital, Bergen, Norway; 8 Centre for International Health, Department of Global Public Health and Primary Care, University of Bergen, Bergen, Norway; 9 Department of Occupational Medicine, Haukeland University Hospital, Bergen, Norway; 10 National Research Centre for the Working Environment, Copenhagen, Denmark

**Keywords:** allergic rhinitis, microbial diversity, microbial exposure, nasal symptoms, pets, place of upbringing

## Abstract

**Background:**

The protective effect of farm upbringing on allergic rhinitis is well known, but how upbringing in other environments influences the development of allergic rhinitis is scarcely investigated. The aim of this study was to investigate the association between place of upbringing and pet keeping in childhood and allergic rhinitis and nasal symptoms in adulthood.

**Methods:**

The population-based Respiratory Health in Northern Europe study includes subjects from Denmark, Norway, Sweden, Iceland, and Estonia born in 1945–1973. This paper analyses 13,376 participants of the third study wave. Six categories of place of upbringing were defined: farm with livestock, farm without livestock, village in rural area, small town, city suburb, and inner city. Pets in the home at birth and during childhood were recorded. Data were analysed using adjusted logistic regression models.

**Results:**

Livestock farm upbringing predicted less adult allergic rhinitis [odds ratio (OR) 0.68, 0.54–0.85] and nasal symptoms (OR 0.82, 0.68–0.99) than city upbringing, and an urban–rural gradient with decreasing risk per level of urbanisation was observed (OR 0.92, 0.88–0.94). Pets in the home at birth (OR 0.78, 0.68–0.88) and during childhood (OR 0.83, 0.74–0.93) were associated with less subsequent allergic rhinitis. Pet keeping did not explain the protective effect of place of upbringing.

**Conclusion:**

Risk of allergic rhinitis and nasal symptoms in adulthood was inversely associated with the level of urbanisation during upbringing. Pets at birth decreased the risk further, but did not explain the urban–rural gradient. Persistent beneficial effects of microbial diversity in early life might be an explanation for the findings.

The prevalence of allergic diseases has increased rapidly since the mid-20th century and has become a major public health problem, particularly in modern industrialised countries (1–3). Allergic rhinitis is the most common of all allergic diseases, and it severely affects the quality of life (4). Allergic rhinitis also represents a substantial economic burden for both individuals and the society (5).

Several studies have shown that the prevalence of allergic rhinitis is lower among people born and raised on a farm (6–9). The microbial load and diversity accompanied with farm living has been suggested as one of the most important reasons for the beneficial effects on allergy (10). However, it is still unclear whether this effect is also present in other environments where the microbial load and diversity may be different.

Elholm et al. showed that the risk of allergic sensitisation and allergic rhinitis in adulthood was gradually decreasing across four levels of upbringing ranging from city to farm (11). However, the study only included inhabitants from Denmark (11). The Danish study confirmed results from a Swedish study also using four categories of urbanisation among adults between 16 and 75 years of age (12), and similar results were suggested in a Finnish study using three categories of urbanisation (13). Recently, an international study with three levels of urbanisation also suggested an urban–rural gradient in allergic rhinitis (14). In the current international study, we included six categories of urbanisation and explored the association with both allergic rhinitis and nasal symptoms, taking pet keeping into consideration.

We hypothesise that the prevalence of allergic rhinitis and nasal symptoms is negatively associated with microbial load and diversity in childhood. To investigate this hypothesis, we used place of upbringing as a proxy for microbial load and diversity in early life and also took pet keeping into consideration.

## Materials and methods

### Study population

This study is conducted on a subpopulation of the European Community Respiratory Health Survey (ECRHS). During recruitment in 1989–1992, the original study population was randomly selected from 22 countries (15, 16). ECRHS Stage 1 included over 130,000 men and women in the age range of 20–44 years (15, 16).

The Respiratory Health in Northern Europe (RHINE) study comprises 21,659 ERCHS subjects from Northern Europe: Norway, Sweden, Iceland, Estonia and Denmark (17). In 1999–2001, a postal questionnaire (RHINE II) was sent out, and 16,105 subjects (74%) responded. At follow-up in 2010–2012 (RHINE III), 13,499 subjects (62%) responded to the postal questionnaire. At both follow-ups, two reminders were sent out (16). The ethics committees for research approved the study for each study centre, and informed consent was obtained from all the study subjects.

### Definition of outcomes, exposures and covariates

Outcomes were derived from RHINE III using the following questions: ‘Do you have any nasal allergies including hay fever?’ and ‘Have you ever experienced nasal symptoms such as nasal congestion, rhinorrhoea (runny nose) and/or sneezing attacks without having a cold?’.

Place of upbringing and pets at birth and in childhood were used as a proxy for load and diversity of microbial exposure. Information on place of upbringing was derived from RHINE III and defined from the following question: ‘What term best describes the place you lived most of the time when you were under the age of 5 years?’ The response categories were 1) farm with livestock, 2) farm without livestock, 3) village in rural area, 4) small town, 5) suburb of city and 6) inner city.

Information on pets at birth and in childhood were derived from RHINE II using the following questions: ‘When you were born, were any of the following in your home?’ and ‘When you were a child, were any of the following in your home?’, with dog, cat and/or other furry animals as response categories for both questions.

Other covariates were derived from the RHINE III questionnaire and included age, sex, smoking, parental smoking in offspring childhood, parental asthma and household size. These were selected *a priori* on the basis of existing evidence about risk factors for allergic rhinitis. Asthma was not included as a covariate in the analyses in order to avoid overadjustment.

### Statistical methods

Data were analysed using logistic regression models and presented as odds ratios (ORs) with corresponding 95% confidence intervals (CI). To be included in the analyses, the subjects had to answer at least one of the two outcome questions. Additional analyses included test for trend and stratified analysis by year of birth and sex. Age stratification was performed before and after 1960 (median birth year).

All statistical analyses were performed using Stata Version 13.1 (StataCorp LP, College Station, TX, USA).

## Results

Basic characteristics for the study population (*N*=13,376) are shown in Table 1.

**Table 1:** Characteristics of the study population

	Inner city	Suburb of city	Small town	Village in rural area	Farm without livestock	Farm with livestock	All analysed	Missing, *N* (%)
Subjects, *N*	2,086	4,047	3,206	1,905	311	1,821	13,376	0 (0%)
Age in 2011±SD	52.6 (±7.0)	50.8 (±7.1)	51.2 (±7.0)	53.1 (±7.2)	51.6 (±6.7)	54.7 (±6.7)	52.1 (±7.1)	308 (2%)
Sex, *N* (%F)	1,042 (50%)	2,093 (52%)	1,688 (53%)	1,075 (57%)	153 (49%)	1,023 (56%)	7,074 (53%)	16 (1%)
Smoking status								4,249 (31%)
Current smoker, *N* (%)	166 (12%)	323 (12%)	199 (9%)	101 (7%)	20 (10%)	75 (6%)	884 (10%)	
Ex-smoker, *N* (%)	452 (34%)	876 (33%)	698 (31%)	467 (34%)	65 (32%)	394 (32%)	2,952 (32%)	
Never smoker, *N* (%)	719 (54%)	1,492 (55%)	1,365 (60%)	818 (59%)	120 (58%)	777 (62%)	5,291 (58%)	
Parental smoking in childhood								537 (4%)
No parental smoking, *N* (%)	516 (26%)	1,198 (30%)	1,036 (33%)	642 (35%)	92 (30%)	779 (45%)	4,263 (33%)	
Mother smoked, *N* (%)	159 (8%)	313 (8%)	234 (8%)	113 (6%)	26 (9%)	89 (5%)	934 (7%)	
Father smoked, *N* (%)	604 (30%)	1,237 (32%)	886 (29%)	626 (35%)	97 (32%)	637 (37%)	4,087 (32%)	
Both parents smoked, *N* (%)	708 (36%)	1,153 (30%)	937 (30%)	434 (24%)	88 (29%)	235 (13%)	3,555 (28%)	
Parental asthma								76 (1%)
Mother, *N* (%)	186 (9%)	338 (8%)	254 (8%)	142 (7%)	29 (9%)	151 (8%)	1,100 (8%)	
Father, *N* (%)	100 (5%)	194 (5%)	149 (5%)	98 (5%)	19 (6%)	93 (5%)	653 (5%)	
Both parents asthma, *N* (%)	12 (1%)	24 (1%)	13 (1%)	15 (1%)	2 (1%)	10 (1%)	76 (1%)	
Centre								0 (0%)
Aarhus, *N* (%)	430 (18%)	713 (31%)	548 (24%)	319 (14%)	33 (1%)	281 (12%)	2,324 (17%)	
Reykjavik, *N* (%)	350 (18%)	792 (42%)	507 (27%)	80 (4%)	20 (1%)	158 (8%)	1,907 (14%)	
Bergen, *N* (%)	432 (18%)	741 (32%)	621 (27%)	92 (4%)	168 (7%)	284 (12%)	2,338 (18%)	
Gothenburg, *N* (%)	297 (18%)	764 (45%)	275 (16%)	224 (13%)	19 (1%)	117 (7%)	1,696 (13%)	
Umeå, *N* (%)	105 (5%)	166 (9%)	531 (28%)	597 (31%)	34 (2%)	495 (25%)	1,928 (14%)	
Uppsala, *N* (%)	303 (16%)	434 (22%)	525 (27%)	418 (22%)	31 (2%)	213 (11%)	1,924 (14%)	
Tartu, *N* (%)	169 (13%)	437 (34%)	199 (16%)	175 (14%)	6 (1%)	273 (22%)	1259 (9%)	
Pets at birth								3,241 (24%)
Yes	172 (12%)	551 (19%)	466 (19%)	629 (41%)	89 (36%)	1,196 (79%)	3,103 (31%)	
Pets in early childhood								2,570 (19%)
Yes	779 (49%)	1,728 (54%)	1,535 (59%)	1,189 (73%)	185 (74%)	1,391 (90%)	6,807 (63%)	

SD, standard deviation.

Subjects who grew up in a city were more likely to be current smokers and exposed to parental smoking in childhood. Pet ownership was more prevalent among subjects from farms and rural areas. Sex and parental asthma status were similar across the six exposure groups.

Place of upbringing was not equally distributed across centres. Fewer subjects from Umeå were brought up in the inner city, and in both Umeå and Uppsala, fewer subjects were brought up in suburbs. Gothenburg and Tartu stand out when it comes to small towns as being less represented, and the same was true for Reykjavik and Bergen for village in a rural area. On the other hand, Bergen was over-represented among subjects from farm without livestock, and Umeå was over-represented for farm with livestock. Gothenburg and Reykjavik were the two centres with the lowest proportion brought up on farms with livestock.

**Fig. 1:**
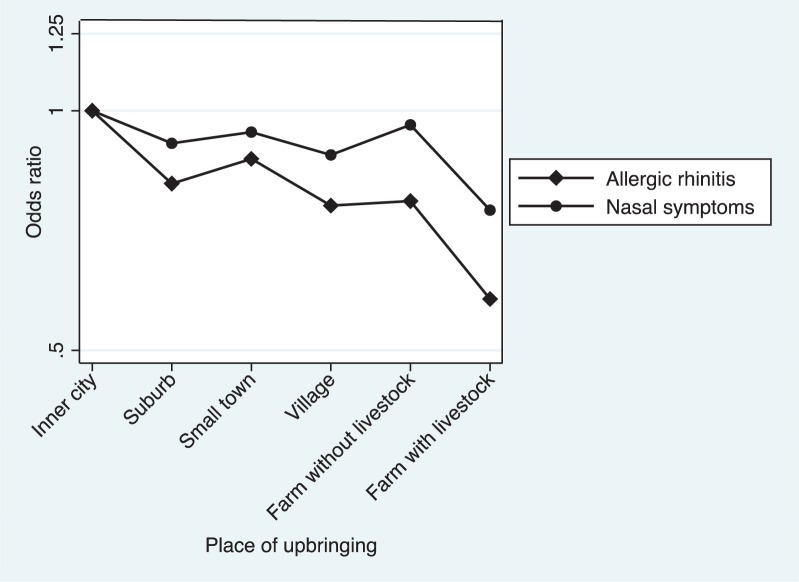
Allergic rhinitis and nasal symptoms in relation to place of upbringing presented as OR adjusted for sex, age, smoking, parental asthma, parental smoking in offspring childhood and centre.

The study population included 3,195 cases with allergic rhinitis and 6,307 cases with nasal symptoms. This corresponds to a prevalence of 24% for allergic rhinitis ranging from 20% on livestock farms to 26% in inner cities, and 47% for nasal symptoms ranging from 45% on livestock farms to 49% in inner cities (Table 2). In adjusted logistic regression models, livestock farm upbringing predicted less adult allergic rhinitis compared with city upbringing (OR 0.58, 0.48–0.71), and a significant decreased risk per level of urbanisation was observed (OR 0.92, 0.88–0.94). Further adjustment for pets and household size resulted in slightly decreased estimates for all six exposure groups. The same pattern was observed for nasal symptoms, although it was less prominent (Fig. 1).

**Table 2:** Logistic regression analyses on allergic rhinitis and nasal symptoms in relation to place of upbringing, OR (95% CI)

	Inner city	Suburb of city	Small town	Village in rural area	Farm without livestock	Farm with livestock	OR for trend^c^
Allergic rhinitis, *N* (%)	538 (26%)	977 (25%)	816 (26%)	438 (24%)	72 (24%)	354 (20%)	
Crude	1	0.91 (0.81–1.03)	0.98 (0.87–1.11)	0.87 (0.7–1.00)	0.88 (0.66–1.17)	0.69 (0.59–0.80)	0.94 (0.92–0.96)
Adjusted 1^a^	1	0.81 (0.69–0.94)	0.87 (0.74–1.02)	0.76 (0.63–0.91)	0.77 (0.54–1.10)	0.58 (0.48–0.71)	0.92 (0.88–0.94)
Adjusted 2^b^	1	0.83 (0.70–0.98)	0.89 (0.75–1.06)	0.82 (0.67–1.00)	0.85 (0.59–1.23)	0.68 (0.54–0.85)	0.95 (0.91–0.98)
Nasal symptoms, *N* (%)	1,013 (49%)	1,904 (48%)	1,560 (49%)	885 (47%)	148 (48%)	797 (45%)	
Crude	1	0.94 (0.85–1.05)	0.99 (0.88–1.10)	0.92 (0.81–1.05)	0.96 (0.75–1.22)	0.83 (0.73–0.95)	0.97 (0.95–0.99)
Adjusted 1^a^	1	0.91 (0.79–1.04)	0.94 (0.82–1.09)	0.88 (0.75–1.04)	0.96 (0.71–1.31)	0.75 (0.63–0.88)	0.95 (0.93–0.99)
Adjusted 2^b^	1	0.94 (0.81–1.09)	1.01 (0.87–1.18)	0.92 (0.77–1.10)	1.05 (0.76–1.45)	0.82 (0.68–0.99)	0.97 (0.94–1.01)

OR, odds ratio.

a Adjusted for sex, age, smoking status, parental asthma, parental smoking status and centre.

b Adjusted for sex, age, smoking status, parental asthma, parental smoking status, centre, family size and pet(s) in the home at birth.

c Comparing two adjacent levels of urbanisation.

Sub-analysis showed no differences between men and women, or between subjects born before and after 1960 (Table 3). No significant interaction between sex or year of birth and place of upbringing was found.

**Table 3:** Adjusted logistic regression analyses on allergic rhinitis and nasal symptoms in relation to place of upbringing stratified by sex and birth year, OR (95% CI).

	Inner city	Suburb of city	Small town	Village in rural area	Farm without livestock	Farm with livestock	OR for trend^c^
Allergic rhinitis							
Men							
Adjusted^a^	1	0.77 (0.62–0.96)	0.79 (0.63–1.00)	0.76 (0.58–1.00)	0.83 (0.40–1.39)	0.50 (0.37–0.69)	0.90 (0.85–0.95)
Women							
Adjusted^a^	1	0.83 (0.66–1.05)	0.94 (0.74–1.19)	0.74 (0.56–0.98)	0.77 (0.46–1.29)	0.65 (0.49–0.86)	0.92 (0.88–0.96)
Born 1945–1959							
Adjusted^b^	1	0.77 (0.62–0.96)	0.76 (0.61–0.95)	0.69 (0.54–0.89)	0.58 (0.33–1.01)	0.59 (0.45–0.76)	0.91 (0.97–0.95)
Born 1960–1973							
Adjusted^b^	1	0.87 (0.70–1.08)	0.99 (0.79–1.23)	0.83 (0.63–1.09)	1.04 (0.64–1.67)	0.53 (0.39–0.74)	0.92 (0.87–0.97)
Nasal symptoms							
Men							
Adjusted^a^	1	0.88 (0.72–1.07)	0.84 (0.69–1.03)	0.92 (0.72–1.16)	1.11 (0.72–1.72)	0.71 (0.55–0.91)	0.95 (0.91–1.00)
Women							
Adjusted^a^	1	0.93 (0.75–1.15)	1.10 (0.89–1.38)	0.82 (0.65–1.05)	0.85 (0.54–1.34)	0.78 (0.61–0.99)	0.95 (0.91–0.99)
Born 1945–1959							
Adjusted^b^	1	0.98 (0.81–1.19)	0.96 (0.79–1.17)	0.92 (0.74–1.15)	0.90 (0.58–1.39)	0.83 (0.67–1.03)	0.96 (0.93–1.01)
Born 1960–1973							
Adjusted^b^	1	0.85 (0.70–1.04)	0.92 (0.75–1.13)	0.84 (0.66–1.07)	1.08 (0.70–1.68)	0.64 (0.49–0.84)	0.94 (0.90–0.99)

OR, odds ratio.

a Adjusted for age, smoking status, parental asthma, parental smoking in offspring childhood and centre.

b Adjusted for sex, age, smoking status, parental asthma, parental smoking in offspring childhood and centre.

c Comparing two adjacent levels of urbanisation.

Pets at birth was associated with a lower prevalence of allergic rhinitis (22% versus 26%) and nasal symptoms (46% versus 48%). In logistic regression models, pets at birth was associated with a decreased risk of both allergic rhinitis (OR 0.78, 0.68–0.88) and nasal symptoms (OR 0.87, 0.78–0.97) (Table 4). Pets during childhood was also associated with a decreased risk of both allergic rhinitis (OR 0.83, 0.74–0.93) and nasal symptoms (OR 0.90, 0.78–1.05). Similar results were found for cat and dog keeping, respectively (results not shown). The results persisted after mutual adjustment for pets at birth and pets during childhood, respectively. Place of upbringing did not modify the association between allergic rhinitis or nasal symptoms and pets in the home (data not shown).

**Table 4:** Logistic regression analyses on allergic rhinitis and nasal symptoms in relation to pets in the home stratified by sex presented as OR (95% CI)

	Pet(s) at birth	Pet(s) during childhood
	
	Yes	Yes
Allergic rhinitis, *N* (%)	661 (22%)	1,591 (24%)
All		
Crude	0.81 (0.73–0.90)	0.89 (0.81–0.98)
Adjusted^a^	0.78 (0.68–0.88)	0.83 (0.74–0.93)
Men		
Crude	0.73 (0.62–0.85)	0.85 (0.74–0.99)
Adjusted^b^	0.65 (0.53–0.79)	0.75 (0.63–0.89)
Women		
Crude	0.83 (0.72–0.96)	0.85 (0.75–0.98)
Adjusted^b^	0.88 (0.75–1.05)	0.90 (0.77–1.05)
Nasal symptoms, *N* (%)	1,416 (30%)	3,195 (48%)
All		
Crude	0.90 (0.83–0.98)	1.02 (0.90–1.15)
Adjusted^a^	0.87 (0.78–0.97)	0.90 (0.78–1.05)
Men		
Crude	0.92 (0.80–1.05)	1.02 (0.90–1.15)
Adjusted^b^	0.79 (0.67–0.93)	0.90 (0.78–1.05)
Women		
Crude	0.88 (0.78–1.00)	0.92 (0.82–1.03)
Adjusted^b^	0.92 (0.79–1.06)	0.94 (0.82–1.08)

a Adjusted for sex, age, smoking status, parental asthma, parental smoking status and centre.

b Adjusted for age, smoking status, parental asthma, parental smoking status and centre.

**Fig. 2:**
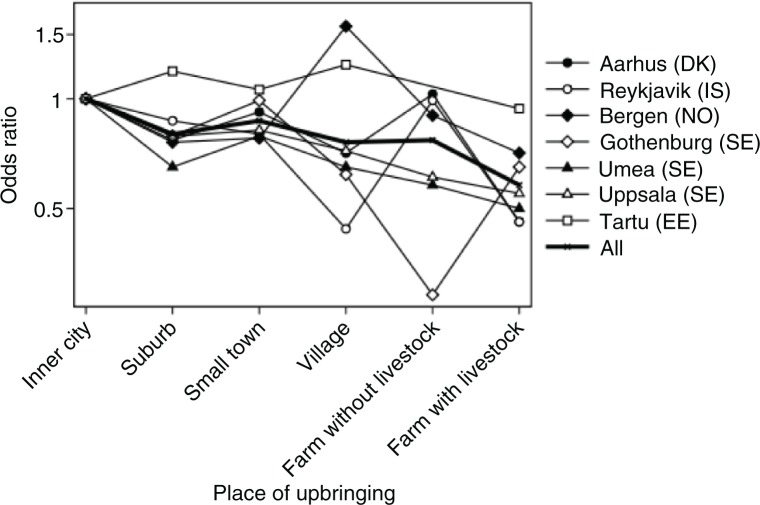
Allergic rhinitis in relation to place of upbringing stratified by study centre adjusted for sex, age, smoking, parental asthma and parental smoking in offspring childhood.

In general, all centres showed an urban–rural gradient. Figure 2 suggests variation between centres, especially for village and for farm without livestock, but no significant difference between centres was revealed (*p*=0.88 for interaction between centre and place of upbringing). For all centres, a decreased risk per level of urbanisation was observed, although not statistically significant for all centres: Aarhus (OR 0.88, 0.82–0.96), Reykjavik (OR 0.86, 0.78–0.95), Bergen (OR 0.98, 0.90–1.08), Gothenburg (OR 0.91, 0.81–1.03), Umeå (OR 0.88, 0.81–1.14), Uppsala (OR 0.90, 0.50–0.81) and Tartu (OR 0.97, 0.86–1.08).

## Discussion

In this population-based study, the risk of allergic rhinitis and nasal symptoms was gradually decreasing across six upbringing levels of urbanisation ranging from inner city to livestock farm. The results were most pronounced for allergic rhinitis. As expected, subjects living on a farm with livestock the first 5 years of their life had significantly less allergic rhinitis and nasal symptoms compared to those living in the inner city. Having pets in the home at birth and during childhood reduced the risk of allergic rhinitis later in life. For nasal symptoms, the protective effect was only statistically significant for pets in the home at birth. Pet keeping did not explain the effect of place of upbringing on allergic rhinitis or nasal symptoms.

Our results are consistent with the findings of Elholm et al., showing an urban–rural gradient for allergic sensitisation and allergic rhinitis among young Danish adults (11), and also support earlier findings from Sweden (12) and Finland (13). Furthermore, an urban–rural gradient has been suggested in the RHINE cohort for inflammatory bowel diseases (18) and asthma (19).

The findings on place of upbringing were comparable across different study centres, although the farming industry and structure differs significantly. In Denmark, relatively big industrialised farms are seen, whereas Norway is known for its smaller farms, often with only one person employed. In addition, Estonia was less urbanised compared to the other countries during the period when the study population was born.

Our findings are not consistent with the findings in the GABRIEL study, where they revealed some centre-specific patterns, that is, the protective effects of farm upbringing on allergic rhinitis were less pronounced in Poland than in Germany, Switzerland and Austria (20).

An important strength of this study is the population-based design with more follow-ups. A limitation of the study is that all variables of interest are self-reported, and therefore a potential risk of recall bias occurs because of the retrospective collection of data. However, we expect that the participants were able to correctly remember their place of upbringing, and therefore we do not consider recall bias to be an issue with the main predictor in our analysis. More critically with regards to recall bias is probably pets in the home at birth. The difficulty is reflected in the low response rate (19–24% missing) for specific question(s) about pet keeping.

In this study, no clinical information about allergic rhinitis was available. This is a clear limitation, and additional analyses on allergic sensitisation would have improved the study. However, a high correlation between immunoglobulin E (IgE)-mediated sensitisation and self-reported allergic rhinitis has been shown in several other studies (21, 22). Our results are more pronounced for allergic rhinitis than for nasal symptoms, which probably reflects that the term ‘nasal symptoms’ is unspecific and covers a range of symptoms. Furthermore, the temporally presence of outcomes differs between the two questions, as the question about allergic rhinitis is on present allergies and the question about nasal symptoms is on ever symptoms.

All centres used a random sample recruitment strategy for including participants. The recruitment strategy may however have varied between the five countries (23).

Only 53% of the original study population responded to all three questionnaires (17). However, a non-response analysis for the three follow-up waves did not show any significant variation in associations between selected exposures (e.g. smoking) and outcomes (e.g. allergic rhinitis) at baseline between long-term responders and only baseline responders (17).

This study only provides information about place of residence for the first 5 years of life. Douwes et al. have suggested that farming exposure might be protective even after 5 years of age when it comes to the development of allergic rhinitis (24). The strongest protection was seen in adults with current and childhood exposure, although the childhood exposure was the strongest protective factor (24). This was supported by Elholm et al. (25).

In this study, we hypothesised that rural upbringing with high microbial load and diversity protects against development of allergic rhinitis and nasal symptoms. It could also be argued that exposures more prevalent in urban areas, for example, air pollution, could explain our findings, and it has been suggested that allergic rhinitis is more common in urban areas with more traffic pollution (26). Even though we would not expect the air pollution level to differ across all six urbanization levels, for example between farm with and farm without livestock, we cannot preclude that others exposures but microbial load and diversity can explain our findings.

It has been argued that allergic rhinitis has a strong genetic component (27), and the results from this study can be explained by selection towards urbanised environments for parents with allergic diseases. In this study, parental asthma status was used as a proxy for the genetic predisposition as data on parental allergic rhinitis were not available. However, adjusting for parental asthma did not change the results. In a recent study by Eduard et al., no difference in asthma prevalence was found among farming apprentices and their non-farmer siblings, which suggests that selection into farming is not substantial (28). In line with that, a study in 1999 found that early farm exposure protects against allergic rhinitis and that it was rather unlikely that it was caused by selection bias from the atopic status of the parents (7). Conversely, a Swedish register study with a large study population showed that subjects with asthma and atopic diseases more often chose to move from farms compared with those without asthma and atopic diseases (29).

In this study, place of upbringing in early childhood is used as a proxy for the microbial load and diversity. However, the study gives no information about how much time the children spent in the stables, at the hayloft, etc. Riedler found that the time spent in the stables plays a dominant role for the protective effect on allergic rhinitis later in life (30). This indicates that the microbial exposure itself is important for the protection against allergic rhinitis and not the livestock farm *per se* as suggested by others (8, 31).

On the other hand, data from the GABRIEL study among children from Switzerland, Austria and Southern Germany indicated that the protective effect against allergic rhinitis was only seen for subjects exposed to livestock animal sheds, haylofts and raw milk in early childhood, but still they found less allergic rhinitis among children growing up on a farm without contact to livestock compared with those growing up in a rural area but not on a farm (32).

Farm children differ in many ways from children growing up elsewhere, which raise the question whether the protective effects are because of other coexisting factors. Strachan et al. argued that the number of (especially older) siblings played a protective role for the development of allergic diseases (33), which has been confirmed by others (34). Farm children in this study and other studies have more siblings than children growing up in urban areas (35–38). However, we adjusted for household size, and this did not change our results.

Pet exposure in early childhood is also an important factor to take into consideration when investigating allergic rhinitis and nasal symptoms. It is debated whether pet exposure is a protective factor or a risk factor for the development of allergic rhinitis (37, 39). We found pet exposure in early life to prevent allergic rhinitis and nasal symptoms, but furthermore we confirmed the results from Elholm et al., who also found an independent effect of place upbringing after adjusting for pet exposure (11).

## Conclusion

Subjects growing up on a farm the first 5 years of life had significantly less allergic rhinitis and nasal symptoms compared with subjects growing up in the inner city. The risk of allergic rhinitis and nasal symptoms was gradually decreasing across each level of urbanisation, resulting in a statistically significant urban–rural gradient. Having pets during childhood protected against the development of allergic rhinitis and nasal symptoms later in life, but did not explain the effect of place of upbringing. These results suggest that early childhood exposures associated with the level of urbanisation play a persistent role for allergic rhinitis and nasal symptoms later in life. Further understanding of exposures associated with urbanisation may contribute to the future prevention of allergic diseases.
